# Biocontrol Efficacy and Genomic Basis of Endophytic Bacteria Against *Xanthomonas campestris* pv. *campestris* in Cabbage

**DOI:** 10.3390/life16040647

**Published:** 2026-04-11

**Authors:** Utku Sanver

**Affiliations:** Department of Plant Protection, Faculty of Agriculture, Siirt University, 56100 Siirt, Türkiye; utkusanver@siirt.edu.tr

**Keywords:** biological control, endophytes, *Pseudomonas synxantha*, *Xanthomonas campestris* pv. *campestris*

## Abstract

*Xanthomonas campestris* pv. *campestris* (Xcc) is the causal agent of black rot, one of the most destructive bacterial diseases on crucifer crops, resulting in yield losses of up to 90%. The aim of this study was to identify novel endophytic bacteria from cabbages with potential biocontrol agents against Xcc. A total of sixty-five isolates were evaluated for plant growth-promoting characters and antagonistic activity, from which ten were selected for *in planta* assays and subsequently validated under field conditions. *Pseudomonas synxantha* BR25/2 consistently demonstrated the highest efficacy, reducing disease severity by 81.12% in *in planta* trials and 33.5% in field trials, thereby comparing to copper-based control measures. Additionally, *Pseudomonas synxantha* BR25/2 significantly enhanced yield parameters, including a 31.8% increase in head weight under field conditions. Whole-genome sequencing identified biosynthetic gene clusters, including siderophores, phenazines, and non-ribosomal peptide synthetases, notably a coronatine-like NRPS and a fengycin-like betalactone, suggesting an extensive antimicrobial potential of metabolites. This represents the first report of *P. synxantha* exhibiting control over Xcc. For commercial application, large-scale fermentation and encapsulation techniques are recommended to overcome shelf-life challenges, providing a sustainable microbial solution for crucifer production.

## 1. Introduction

Crucifer crops, including cabbage, broccoli, kale, cauliflower, and brussels sprouts, serve as important constituents of global agriculture, attributed to their nutritional value and economic importance. In 2020, the global production of vegetable crucifers reached 96.4 million tons, reflecting a significant increase of 10.6% over the preceding decade [1]. These vegetables are well documented for their elevated concentrations of essential nutrients and health-promoting phytochemicals, including phenolics, glucosinolates, vitamins, and minerals. Experimental evidence suggests that a higher intake of crucifer foods is linked to a decreased risk of degenerative diseases, cancer, cardiovascular diseases, and immune disorders [2].

However, the cultivation of crucifers presents several challenges. Pathogens like black rot, clubroot, downy mildew, and turnip yellows virus can importantly affect crop yields, with losses potentially stretching as high as 90% in certain instances [1]. The most destructive of these pathogens is *Xanthomonas campestris* pv. *campestris* (Pammel 1895) Dowson 1939 (Xcc), a significant bacterial pathogen responsible for black rot in crucifer crops, including economically important species such as cabbage, broccoli, and rapeseed [3]. This pathogen is recognized for its devastating effect on global agriculture, making it a critical topic of inquiry in the field of plant pathology. Xcc is a rod-shaped, Gram-negative bacterium classified within the γ-subdivision of Proteobacteria, first described in 1889 [4]. The pathogen’s capacity to survive in diverse environments, comprising soil, seeds, and plant residues, contributes to its persistence and dissemination [5]. Xcc is diagnosed as a seed-transmitted vascular disease agent that mainly infiltrates plant tissues via hydathodes, specialized structures located at the leaf margins [6]. Young crucifer plants are particularly vulnerable to Xcc, with infections leading to reduced biomass and photosynthesis. This vulnerability demands increased disease control measures during the early stages to prevent losses in adult plants. Interestingly, adult plants exhibit a better immune response, and no important loss is observed when they are infected [7]. For example, in cauliflower, black rot can lead to yield losses ranging from 10% to 50%, affecting both curd quality and seed vigor. The disease severity in cauliflower can be as high as 100% in susceptible varieties, with significant effects on yield [8]. It is characterized by a complex species race division and noteworthy similarities to other species within the Xanthomonas genus, which pose difficulties for accurate diagnosis [9]. The bacterium’s persistence in soil and plant residues additionally exacerbates its threat in agricultural contexts [10]. Xcc employs a type III secretion system (T3SS) to translocate effector proteins into host cells, thereby facilitating infection and undermining plant immunity [4]. During the first stages of infection, Xcc adapts to the plant environment by upregulating genes associated with metabolic adaptations and virulence factors [6].

Several strategies have been explored to control Xcc. A critical control method is the application of seed treatments, particularly for seed-borne disease agents such as Xcc. For instance, hot water treatment at 50 °C for 30 min has been shown to importantly reduce the severity of black rot disease by 80.67% in cabbage seedlings [11]. Another effective strategy involves the identification of resistant genotypes, with field trials having identified several resistant and moderately resistant genotypes of rapeseed-mustard that may be utilized in breeding programs to develop cultivars with enhanced resistance [12]. Furthermore, traditional copper-based bactericides are progressively losing their efficacy due to the development of resistance. Consequently, alternative approaches, including plant defense activators such as Actigard and Howler, as well as sulfur-based treatments, have been evaluated. While certain alternatives have demonstrated potential in reducing disease severity, they have not resulted in significant improvements in crop yields [13]. Biological control of Xcc is a potential alternative to chemical treatments. Several studies have explored the use of antagonistic bacteria, bacteriophages, and quorum quenching strategies to control this pathogen strongly. These biological control methods not only reduce reliance on chemical pesticides but also promote sustainable agricultural practices.

Among these biological control strategies, the use of endophytic microorganisms has emerged as a particularly promising approach due to their unique ability to interact directly with the host from within. Endophytic microorganisms reside within the internal tissues of plants for at least part of their life cycle without causing apparent disease symptoms. These microorganisms play a pivotal ecological role by forming symbiotic relationships that enhance host resilience against various biotic and abiotic stresses [14]. From an agricultural perspective, endophytes are of paramount importance as they can promote plant growth through the production of phytohormones, improvement of nutrient acquisition, and induction of systemic resistance (ISR) [15]. Furthermore, their ability to colonize the same ecological niches as vascular pathogens makes them highly effective biocontrol agents [16].

For example, *Bacillus velezensis* Ruiz-García 2005 has shown appreciable antagonistic activity against Xcc. It secretes lipopeptides and siderophores like bacillibactin, which contribute to the inhibition of Xcc by causing cell lysis and death [17]. *Bacillus* spp. have also shown strong inhibitory activities, with some isolates achieving 100% growth inhibition of Xcc in vitro [18]. *Paenibacillus* sp. isolates have been successful in inhibiting Xcc activity, with in vivo assays demonstrating reduced colonization and disease incidence in kale crops [18]. *Pseudomonas* spp., along with *Bacillus* spp., have been identified as strong antagonists against Xcc [19]. Currently, the management of black rot primarily relies on copper-based bactericides. However, the intensive use of these chemical agents has led to significant environmental pollution and the emergence of resistant Xcc strains. Therefore, there is an urgent need to identify novel and sustainable microbial biocontrol agents. This study was carried out to assess the efficacy of novel endophytic bacteria, isolated from plant tissues in cabbage fields, against the Xcc pathogen. The assessment was conducted via in vitro plant growth-promoting rhizobacteria tests and *in planta* assays. The bacterial isolates exhibiting the highest efficacy were subsequently assessed under field conditions to determine their impact on the disease severity and crop yield. Furthermore, a comprehensive genomic analysis of the most successful bacterium was undertaken to explain the mechanisms underlying its efficacy and its novelty as a potential biopesticide.

## 2. Materials and Methods

### 2.1. Collection and Isolation of Samples from the Field

**Figure 1:**
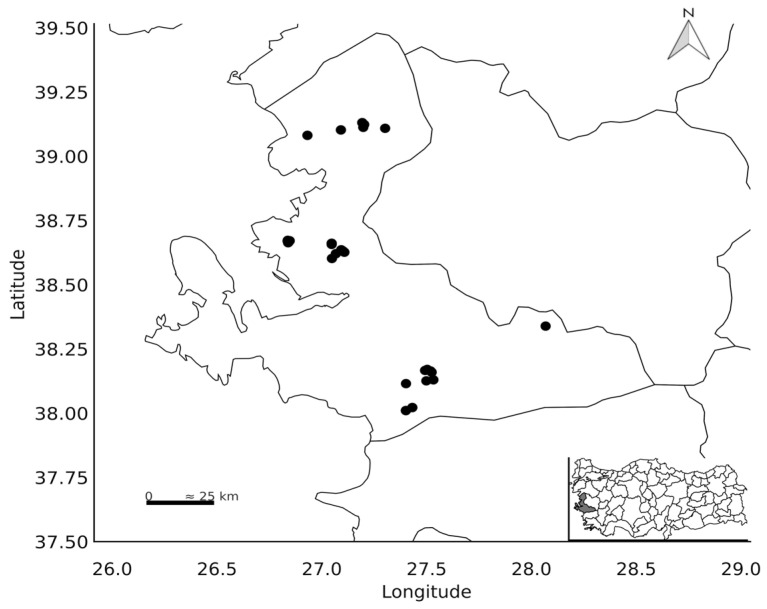
Map illustrating the geographical locations from which cabbage samples were collected.

The study was conducted during the 2023 and 2024 growing seasons. Sampling was carried out in 44 different agricultural fields, representing various ecological regions within the city of İzmir, Turkey (Figure 1). A single representative healthy plant sample was collected from each field. Detailed information concerning the precise GPS coordinates and the characteristics of the isolates obtained is comprehensively documented in Table S1.

Endophytic bacteria were isolated from the leaf, stem, and root tissues of cabbage plant samples obtained from field collections. Surface decontamination of leaf samples was conducted following the protocol established by Ozaktan et al. [20]. Plant samples were initially washed with running tap water, followed by rinsing with distilled water within a laminar airflow hood. Subsequently, the samples were immersed in 70% (*v*/*v*) ethanol for 3 min and then treated with 5% (*v*/*v*) sodium hypochlorite for 5 min. To ensure thorough decontamination, the samples were rinsed four times with autoclaved sterile distilled water. To confirm the effectiveness of the surface sterilization, the final rinsing solution was inoculated onto KingB (KB) medium and incubated at 25 °C for 48 h. Surface-sterilized plant samples were then sectioned into approximately 1 cm^2^ pieces using a sterile scalpel and were subsequently imprinted onto KB medium by applying imprinting. The isolated and purified bacterial strains were transferred to Nutrient Broth medium supplemented with 20% glycerol for subsequent analysis and stored in an ultra-low-temperature freezer at −86 °C (Glacier, NuAire, Inc., Plymouth, MN, USA).

### 2.2. Preliminary Bacterial Diagnosis and In Vitro Assays of Plant Growth-Promoting Rhizobacteria (PGPR)

Isolates were procured from cabbage plants collected in the field. As part of a preliminary screening procedure, these isolates underwent a series of assessments, including the Gram-stain test, evaluation of fluorescent pigmentation, assessment of pectolytic activity, examination of tobacco hypersensitivity reactions, and growth tests conducted at 37 °C, as described by Schaad et al. [21]. In this selection process, isolates showing pectolytic activity or tobacco hypersensitivity reactions were identified as potential plant pathogens, while those exhibiting growth at 37 °C were considered potential risks to human and animal health [21]. Consequently, these potentially pathogenic isolates were excluded from further analysis to ensure the safety of the selected biocontrol agents. The remaining isolates were subsequently evaluated for their in vitro characteristics as plant growth-promoting rhizobacteria (PGPR). In vitro assays of PGPR were conducted to evaluate the indole-3-acetic acid (IAA) production, siderophore production, hydrogen cyanide (HCN) production, phosphate solubilization, and antagonistic activity against Xcc.

IAA production and estimation were conducted according to the methodology described by Gang et al. [22]. The biosynthesis of IAA in the cultures was stimulated by the addition of 0.3% (*w*/*v*) L-tryptophan to the nutrient broth, followed by incubation of the cultures in the dark at 25 °C on an orbital shaker (Unimax 2010, Heidolph Instruments, Schwabach, Germany) set at 120 rpm. IAA production and secretion were assessed in the culture supernatants after a 24 h incubation period using Salkowski reagent. Specifically, 1 mL of culture supernatant was combined with 1 mL of Salkowski reagent and incubated in the dark for 30 min. The development of a pink color was quantitatively measured spectrophotometrically at 535 nm, and IAA concentrations were determined using an IAA standard. The assay was performed in triplicate.

Siderophore production was conducted in accordance with the methodology described by Sarvepalli et al. [23]. Chromium Azurol S (CAS) agar plates were prepared by dissolving 2 mM CAS in 50 mL of distilled water (dH_2_O), followed by the addition of 10 mL L^−1^ of 1 mM ferric chloride and 10 mL L^−1^ of hydrochloric acid. Subsequently, 40 mL of a 72.9 mg L^−1^ solution of hexadecyltrimethylammonium bromide was incorporated, resulting in the formation of a dark blue solution. The final solution was then subjected to autoclaving. A separate mixture was prepared using 800 mL of dH_2_O, 30.24 g L^−1^ of piperazine, 100 mL of production medium, and 20 g L^−1^ of agar, adjusted to a pH of 7.0 ± 0.2; and this mixture was also autoclaved (HVE-50, Hirayama Manufacturing Crop., Kasukabe, Japan). Following autoclaving, this mixture was combined with the previously prepared dye solution while continuously stirring to prevent foam formation, and it was then aseptically transferred into sterile Petri dishes. All bacterial isolates were inoculated onto the CAS agar plates via spot inoculation and incubated at 25 °C for a week. Subsequently, the orange and yellow zones that developed around the bacterial colonies were measured in millimeters and evaluated. All treatments were carried out in triplicate.

Phosphate solubility was evaluated utilizing the National Botanical Research Institute’s Phosphate Growth Medium (NBRIP) [24], as described by Suleimanova et al. [25]. The medium composition included glucose (10 g L^−1^), magnesium chloride hexahydrate (MgCl_2_·6H_2_O, 5 g L^−1^), magnesium sulfate heptahydrate (MgSO_4_·7H_2_O, 0.25 g L^−1^), potassium chloride (KCl, 0.2 g L^−1^), ammonium sulfate ((NH_4_)_2_SO_4_, 0.1 g L^−1^), and distilled water, with the pH adjusted to 7.0 ± 0.2 prior to sterilization. Various sources of phosphorus, including tricalcium phosphate (Ca_3_(PO_4_)_2_), calcium hydroorthophosphate (CaHPO_4_), hydroxyapatite, phosphorite, aluminum phosphate (AlPO_4_), and iron phosphate (Fe_3_(PO_4_)_2_), were employed at a concentration of 5 g L^−1^ as the sole phosphorus source. The solid NBRIP medium was supplemented with 20 g L^−1^ agar. Bacterial strains were inoculated onto the agar plates through dot inoculation and subsequently incubated at 25 °C for a duration of 10 days. Each isolate was tested in triplicate.

A qualitative assessment of hydrogen cyanide (HCN) production by the bacterial isolate strain was conducted utilizing the method described by Verma et al. [26]. The bacterial cultures were inoculated onto King’s B medium, and sterile filter paper, saturated with a picric acid solution (composed of 2.5 g L^−1^ picric acid and 12.5 g L^−1^ sodium carbonate in dH_2_O), was affixed to the lids of three Petri dishes. The Petri dishes were subsequently sealed with Parafilm^®^ and incubated under controlled laboratory conditions for a duration of three days. A transition in the color of the filter paper from yellow to brown was recorded as a positive indicator of HCN production. This assessment was performed to determine the presence or absence of HCN, and the results were visually verified.

The antagonistic activities of bacterial isolates against Xcc were assessed utilizing a dual culture assay, as described by Li et al. [27]. The Xcc strain MS209 (accession number: PX307907) and the candidate bacterial isolates were cultured for 48 h at 25 °C on KB medium. Subsequently, suspensions were prepared to achieve equivalent cell densities for the candidate bacteria (OD_600_ = 1.0) and Xcc (OD_600_ = 0.35). The Xcc suspension was then uniformly spread onto sterile 9 mm diameter Petri dishes employing the spread plating technique. The plates were incubated at 25 °C for a duration of 48 h. The evaluation of antagonistic activity was conducted by measuring the diameter of the growth inhibition zone in millimeters. The dual culture assay was conducted in triplicate to ensure reproducibility.

In vitro test results were assessed utilizing the weighted rating system. For the calculation of the weighted rating system (WRS), coefficients were assigned to each characteristic by the researchers based on their relative importance [28]. In this study, the coefficients were determined as follows: 0.04 for hydrogen cyanide (HCN) production, 0.12 for indole acetic acid (IAA) production, 0.12 for phosphate solubilization, 0.32 for siderophore production, and 0.40 for the dual culture test.

$$WRS=\left[\frac{0.04.\frac{HCN}{a}+0.12.\frac{IAA}{a}+0.12.\frac{PS}{a}+0.32.\frac{Sid}{a}+0.40.\frac{AA}{a}}{0.04.\frac{HCNb}{a}+0.12.\frac{IAAb}{a}+0.12.\frac{PSb}{a}+0.32.\frac{Sidb}{a}+0.40.\frac{AAb}{a}}\right]\times 100$$

The calculation methodology entails the following steps. The numerator of the formula is calculated by dividing the value generated by the isolate for each characteristic by the average value of all isolates exhibiting the same characteristic (denoted as ‘a’), followed by multiplying by the designated coefficient for that category. The denominator is calculated by dividing the highest value (denoted as ‘b’) for each characteristic by the overall average (denoted as ‘a’) for that characteristic, and subsequently multiplying by the corresponding coefficient. The values computed for each characteristic are aggregated to yield a maximum value representing all characteristics. The cumulative values for all characteristics pertaining to an isolate are then summed and divided by the maximum value. The resulting figure is multiplied by 100 to derive the WRS value for that isolate. In this formula, PS represents phosphate solubilization activity, Sid denotes siderophore activity, IAA indicates indole acetic acid production ability, HCN refers to hydrogen cyanide production ability, and AA signifies antagonistic activity against Xcc. The term ‘isolate’s value’ refers to the specific value for that characteristic, ‘a’ is the average value for that characteristic among the isolates (with those exhibiting no activity excluded from the average), and ‘b’ represents the highest value attained for that characteristic among the isolates. Based on these results, the ten isolates demonstrating the highest performance were selected for *in planta* trials.

### 2.3. In Planta Assays for Determining the Efficacy Against Xanthomonas Campestris pv. campestris

Following the in vitro assay for PGPR, ten endophytic bacterial isolates were selected for *in planta* trials based on their weight rating scores against the Xcc pathogen. The test plant utilized in these trials was cabbage (*Brassica oleracea* var. *capitata* f. *alba*) cv Super Pamir F1, which is known to be susceptible to the Xcc pathogen. This variety is characterized as a flat, compact, soft, thin-leaved white cabbage suitable for both spring and autumn cultivation, with a growing period of 80 to 85 days and an average head weight ranging from 3 to 4 kg [29]. The pathogen employed in this study was the Xcc isolate MS209 (accession number: PX307907), which is part of the bacterial culture collection at the Plant Protection Department of the Faculty of Agriculture, Ege University. Cabbages were initially developed in trays and subsequently transplanted into 9 × 9 × 8 cm plastic pots upon reaching the three-true-leaf stage. The growing medium used was KLASSMANN TS1 peat, which exhibited an electrical conductivity of 35 mS m^−1^, a pH value of 6.5, a nutrient composition of 14:10:18 (N:P:K), and a density of 1.0 kg m^−3^ [30].

For the *in planta* experiment, the selected bacterial isolates were inoculated into KingB medium. After incubating for 48 h, the bacterial cultures were utilized to prepare a suspension with a density of OD_600_:0.1 (1 × 10^8^ CFU mL^−1^), as measured using a UV-VIS spectrophotometer (T60, PG Instruments Ltd, lutterworth UK). This suspension was then applied to the plant surface using a hand sprayer. Two days post-inoculation, the Xcc pathogen was introduced to the plants. To this end, bacteria cultured in King medium for 48 h were prepared into a suspension with an OD_600_:0.35 (1 × 10^8^ CFU mL^−1^) concentration, again utilizing a UV-VIS spectrophotometer. This suspension was inoculated into the hydathode region via a spray application, ensuring that approximately 0.5 mL of the suspension was delivered per plant. The plants were subsequently maintained in a growth chamber under controlled conditions, featuring a 16:8 light–dark cycle, 70% humidity, a temperature of 25 °C, and a light intensity of 10,000 lux. Disease assessments were conducted three weeks following the inoculation.

Disease severity was evaluated utilizing a scale adapted from the 0–4 criteria established by Peňázová et al. [31]. The severity classifications are as follows: 0: 0% necrotic area on leaf; 1: necrotic area on leaf <25%; 2: necrotic area on leaf 25–50%; 3: necrotic area on leaf 50–75%; and 4: necrotic area on leaf >75%. The scale values obtained were converted to percentage disease severity index (DSI %) using the Townsend–Heuberger formula [32].

$$DSI\;(\%)=\frac{\Sigma (n\times v)}{\left(N\times V\right)}\times 100$$

In this formula, ‘n’ represents the number of leaves in each category, ‘v’ is the numerical value assigned to each category based on the disease scale, ‘N’ is the total number of leaves examined, and ‘V’ is the maximum value of the scale used. The biocontrol efficacy was calculated from the Percent Disease Index (DSI %) using the Abbott formula [33]:

$$Efficacy\;(\%)=\frac{\left(C-T\right)}{C}\times 100$$

In this formula, C represents the Percent Disease Severity Index in the control untreated plants, and T represents the Percent Disease Index in the treated plants. This calculation allowed for a standardized comparison of the performance of the isolates against Xcc.

*In planta* assays were established in ten replicates for each parameter, and the assays were conducted three times. Subsequent to the *in planta* assay, the two most effective isolates were selected for the field trial.

### 2.4. Determination of the Effects of Xanthomonas Campestris Pv. Campestris and Plant Yield Under Field Conditions

Field trials were conducted from August to November 2025 at two distinct experimental sites in Izmir, Turkey, to assess the efficacy of biological control under varying soil conditions while maintaining consistent climatic parameters. Field 1 (38.451560 N, 27.225593 E) exhibited a loamy soil structure, characterized by relatively lower soil moisture levels and enhanced aeration. Conversely, Field 2 (38.450692 N, 27.218699 E) was distinguished by its clay-rich soil composition, which afforded greater water-holding capacity and diminished aeration. Gübretaş BestStarter N:P:K 13:18:5 + 2(MgO) + 10(SO_3_) base fertilizer was applied to the tilled land. The Pamir F1 variety of cabbage was used. Inoculations with bacteria and disease agents were performed. The commercial control was Cu_2_(OH)_3_Cl (Copper oxychloride 700 g L^−1^; Sygenta Cuprocol), and the seedlings were transplanted to the field trial area. The cabbage seedlings were planted 70 cm apart in rows with 50 cm between each row.

The application protocol described in detail in the *in planta* study was followed. The treatments applied in the field trial were delineated as follows: 1. Isolate 1, 2. Isolate 2, 3. Cu_2_(OH)_3_Cl, 4. untreated control, 5. Isolate 1 + Xcc, 6. Isolate 2 + Xcc, 7. Cu_2_(OH)_3_Cl + Xcc, 8. untreated control + Xcc. The field trials were conducted in four replicates, with a plant per replicate, following a randomized complete block design. Following the establishment of the trial, disease progression was monitored biweekly for a duration of three months. Disease severity was evaluated using the 0–4 scale developed by Penazova et al. [31], which was previously detailed in the *in planta* assay section. Each individual leaf of the four replicated plants per treatment was scored separately according to the scale. The obtained scale scores for each plant were converted into a percentage of disease severity index using the Townsend–Heuberger formula. The cabbage heads harvested after three months were subjected to measurements of fruit weight, length, width, vertical diameter, and horizontal diameter for the purpose of yield assessment.

### 2.5. Molecular Analysis Conducted on the Most Successful Bacterial Strains

The bacteria identified as most successful at the conclusion of the *in planta* trial were characterized through 16S Sanger sequencing. Subsequently, whole-genome analysis utilizing Illumina sequencing was conducted to elucidate the presence of metabolite genes associated with the bacterium taken for most successful at the conclusion of the field trial. In preparation for DNA extraction, bacteria were cultivated on KB medium for a duration of 24 to 48 h at a temperature of 24 °C. DNA was extracted utilizing the ThermoFisher DNA purification kit, resulting in a concentration of 50 ng/μL and an A260/280 ratio ranging from 1.8 to 2.0. The extracted DNA served as a template for polymerase chain reaction (PCR) amplification for 16S sanger sequencing [34]. Universal primers 27F (5′ AGAGTTTGATCMTGGCTCAG 3′) and 1492R (5′ TACGGYTACCTTGTTACGACTT 3′) were employed in the PCR to amplify approximately 1500 base pairs of 16S rRNA [35]. The final PCR mixture comprised 100 ng of DNA extract, 10× Taq KCl reaction buffer, 1 mM of each primer, 1.5 mM MgCl_2_, 0.2 mM dNTP, and 1 U of Taq DNA polymerase (recombinant 5 U/μL). The amplification protocol consisted of an initial denaturation at 95 °C for 3 min, followed by 35 cycles of denaturation at 94 °C for 1 min, annealing at 50 °C for 1 min, and extension at 72 °C for 2 min, concluding with a final extension at 72 °C for 5 min, all conducted in a thermal cycler (Mastercycler 5332, Eppendorf AG, Hamburg, Germany). The resulting PCR products underwent electrophoresis on a 1.5% agarose gel in 0.5× TAE buffer (50× Tris-acetate-EDTA) supplemented with RedSafe™ nucleic acid staining solution (20,000×). The gel was operated at 80 V for 90 min. DNA bands were visualized under ultraviolet light employing a GeneRuler™ 1 kb DNA Ladder. The PCR products were subsequently sent to MedSantek company for Sanger sequencing. Sequence products were assembled into contigs and analyzed using BLASTn software (v2.16.0).

Following the isolation of DNA, the concentration of the samples was measured at 50 ng/µL, and their purity was confirmed with an A260/280 ratio of 1.8. The DNA samples taken for suitable were subsequently sent to Genoks Genetic Center Company for whole-genome sequencing (WGS). The raw data acquired in FASTQ format as a result of sequencing was analyzed using FASTQC (v 0.12.1) [36] to ensure quality control. Trimming operations were conducted with Trimmomatic (v 0.39) [37] to eliminate low-quality base calls and adapter contamination. The data that met the quality control standards was subjected to de novo genome assembly utilizing Shovill (v 1.4.2) [38], resulting in the generation of contig sequences. These contig sequences were further evaluated with antiSMASH (v 6.1.1) [39] to assess the potential for antimicrobial metabolite biosynthesis, and the relevant biosynthetic gene clusters (BGCs) were annotated. Finally, the assembly results were visualized using pyCirclize (v 0.7.0) [40].

### 2.6. Statistical Analysis

The in vitro assays were performed in a completely randomized design (CRD) with three replicates, while the *in planta* experiments were conducted in a CRD with 10 replicates per treatment. The field trials were laid out in a randomized complete block design with four replications. The *in planta* assays and field trial experiments conducted in this study were analyzed with a one-way analysis of variance (One-Way ANOVA) alongside a multiple comparison test at a 95% confidence level, specifically the Tukey test. Core libraries within R were employed for the correlation matrix and PCA biplot analyses. Additionally, the ‘ggplot2’ was utilized for the graphical visualization of the results.

## 3. Results

### 3.1. Subsection Results of In Vitro Assay

**Figure 2:**
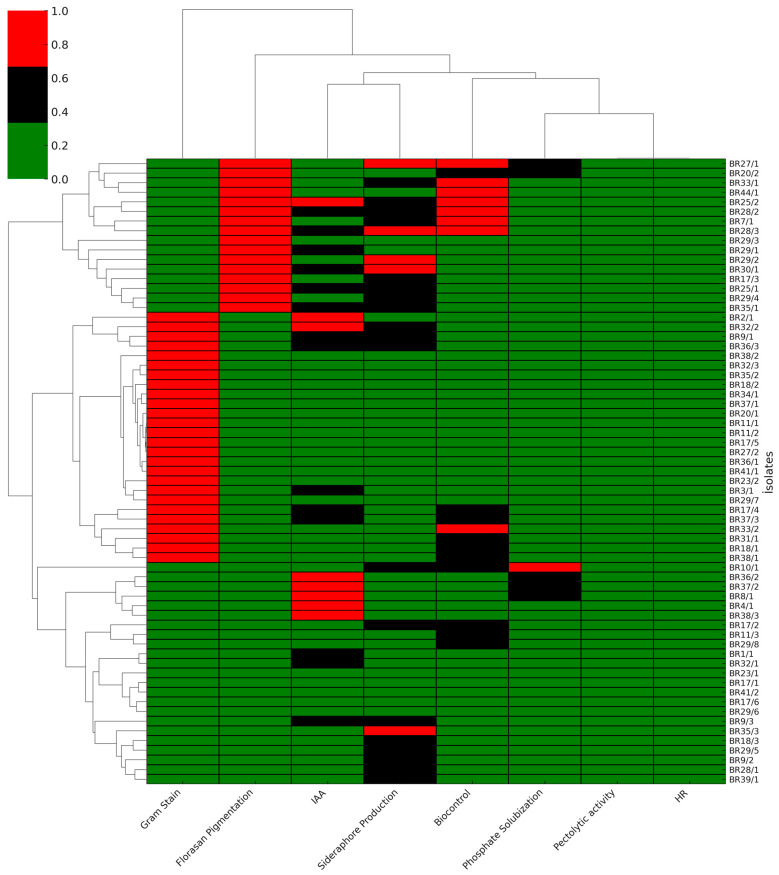
Hierarchical cluster analysis and heatmap representation of the in vitro functional profiles of the bacterial isolates. The vertical dendrogram represents the similarity relationships among isolates based on their PGP and biocontrol traits, while the horizontal dendrogram clusters the evaluated parameters.

A total of 65 bacterial isolates, obtained from several locations, were assessed for their biochemical and PGPR-related characteristics. Gram staining results indicated that 40.0% (26 isolates) were Gram-positive, while 60.0% (39 isolates) were Gram-negative, indicating a predominance of Gram-negative isolates. Analyzing the distribution by location, the highest proportion of isolates was identified in Menemen (33.3%, 22 isolates), followed by Foça (16.9%, 11 isolates), Bergama (15.4%, 10 isolates), Selçuk (13.8%, 9 isolates), Bayındır (9.2%, 6 isolates), Torbalı (4.6%, 3 isolates), and Dikili (3.1%, 2 isolates).The lowest numbers were recorded in Ödemiş (1.5%, 1 isolate) and Kınık (1.5%, 1 isolate). Regarding PGPR traits, siderophore production was observed in 75.4% (49 isolates), with BR29/2 (19.86 mm), BR28/3 (17.03 mm), and BR30/1 (14.37 mm) identified as the most efficient producers. Phosphatase activity was detected in 27.7% (18 isolates), with the highest activity noted in BR10/1 (8.92 mm), BR36/2 (4.12 mm), and BR37/2 (5.18 mm). All isolates (100%, 65 isolates) exhibited positive results for IAA production, with BR37/2 (53.30 µg mL^−1^), BR36/2 (52.55 µg mL^−1^), BR38/3 (48.79 µg mL^−1^), BR32/2 (44.84 µg ml^−1^), and BR25/2 (41.45 µg mL^−1^) demonstrating the highest concentrations. Notably, none of the isolates produced hydrogen cyanide (HCN) (0.0%). In vitro biocontrol activity was observed in 32.3% (21 isolates), with the most pronounced antagonistic effects attributed to BR33/2 (9.93 mm), BR44/1 (9.19 mm) and BR7/1 (8.55 mm) (Table S1, Figure 2).

Based on the weight rating scores, the isolates BR28/3, BR27/1, BR7/1, BR27/2, BR10/1, BR44/1, BR33/2, BR28/2, BR25/2, and BR34/1 were selected for the *in planta* testing procedure.

### 3.2. Results of in Planta Assays

To further validate the antagonistic potential of the candidates, the biocontrol efficacy of the ten selected isolates was tested *in planta*, with the resulting data presented in Table 1.

In the first *in planta* assay, the analysis of variance (ANOVA) indicated significant differences among the treatments (*p* = 2.1 × 10^−8^). The untreated group presented a disease severity of 38.23 ± 3.86% (0% efficacy). According to the Tukey test, BR25/2 emerged as the most effective isolate, exhibiting a severity of 10.82 ± 4.07% (71.70% efficacy). This was followed by BR44/1 (14.32 ± 4.07%; 62.54% efficacy) and BR27/2 (15.66 ± 4.07%; 59.04% efficacy), both of which significantly mitigated disease symptoms relative to the untreated group.

In the second *in planta* assay, ANOVA again revealed statistically significant variations (*p* = 0.00445). The disease severity in the untreated group was recorded at 27.81 ± 3.86%. BR25/2 demonstrated the highest efficacy (9.92 ± 4.07%; 64.33% efficacy), followed by BR34/1 (11.21 ± 3.86%; 59.69% efficacy) and BR27/1 (12.84 ± 3.86%; 53.83% efficacy). Moderate protection was observed in isolates BR10/1 and BR33/2, whereas BR7/1 (18.66 ± 4.62%; 32.90% efficacy) exhibited the least effectiveness.

In the third trial, characterized by elevated disease pressure, ANOVA confirmed significant differences (*p* = 2.2 × 10^−16^). The untreated group reached a severity of 81.37 ± 3.29%. Under these conditions, BR25/2 (6.82 ± 3.68%; 91.62% efficacy) and BR10/1 (14.00 ± 3.29%; 82.79% efficacy) emerged as the most resilient treatments. Additional isolates, including BR28/2 (9.26 ± 3.29%; 88.62% efficacy) and BR44/1 (10.27 ± 3.94%; 87.38% efficacy), also provided substantial protection.

**Figure 3:**
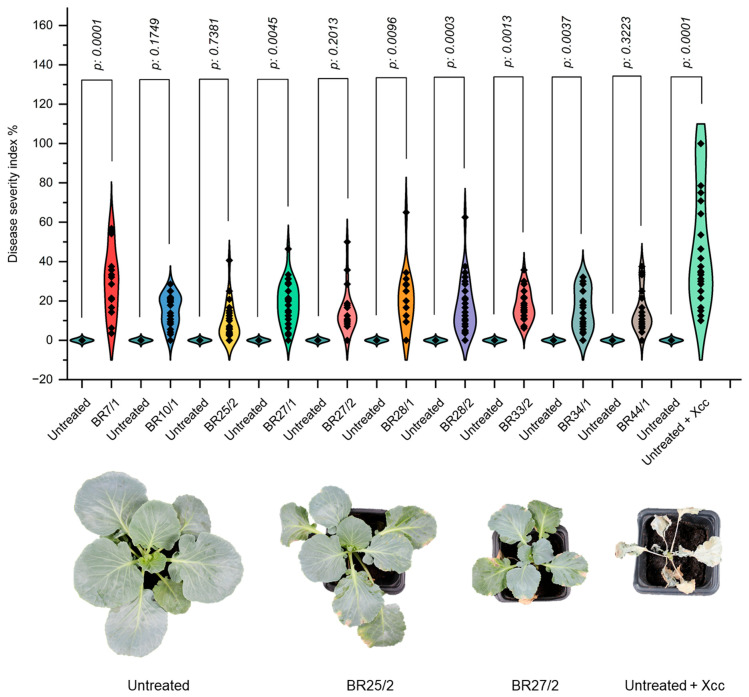
*In planta* biocontrol efficacy of endophytic isolates against Xcc. The upper row illustrates violin plots showing disease severity index (%) based on Townsend–Heuberger conversion, with exact *p*-values for statistical significance. The lower row represents visual impact of selected isolates on black rot symptoms compared to controls.

The overall assessment across all trials (Figure 3) indicated that BR25/2 was the most consistent isolate, with a mean severity of 9.28 ± 2.77% (81.12% efficacy). Subsequently, BR27/2 (71.14% efficacy) and BR44/1 (70.49% efficacy) were also recognized for their commendable performance. Based on these results, BR25/2 and BR27/2 were selected for further field trials.

### 3.3. Results of in Field Trials

#### 3.3.1. Results of Disease Progression in Field Trials

The field trials conducted at two distinct locations demonstrated that the application of endophytic isolates significantly suppressed the progression of Xcc compared to the positive control.

In the first field trial, no disease symptoms were initially observed; however, significant differences among treatments emerged by the second week (*p* = 0.147). At this early stage, BR25/2 + Xcc (6.25 ± 6.25%; 75.0% efficacy) and the chemical control Cu_2_(OH)_3_Cl + Xcc (6.25 ± 6.25%; 75.0% efficacy) exhibited the most effective suppression. By the midpoint (Week 6), disease pressure in the untreated + Xcc group increased to 70.00 ± 7.14%, while BR25/2 + Xcc maintained a significantly lower severity (37.50 ± 6.75%; 46.4% efficacy). At the conclusion of the trial (Week 12), the ANOVA revealed highly significant differences between groups (*p* = 0.029), with BR25/2 + Xcc (59.06 ± 11.81%; 33.5% efficacy) consistently outperforming BR27/2 + Xcc and the chemical treatment, categorizing it in a distinct statistical group (‘b’).

The second field trial exhibited a similar statistical trend. As disease pressure peaked towards the end of the season (Week 10), BR25/2 + Xcc (53.75 ± 4.27%; 21.5% efficacy) and the chemical control (58.65 ± 6.19%; 14.3% efficacy) continued to exhibit statistically significant reductions in disease severity compared to the positive control (*p* = 0.029). By Week 12, BR25/2 + Xcc (58.75 ± 3.92%; 19.3% efficacy) retained its status as the most stable biocontrol candidate (*p* = 0.409).

**Figure 4:**
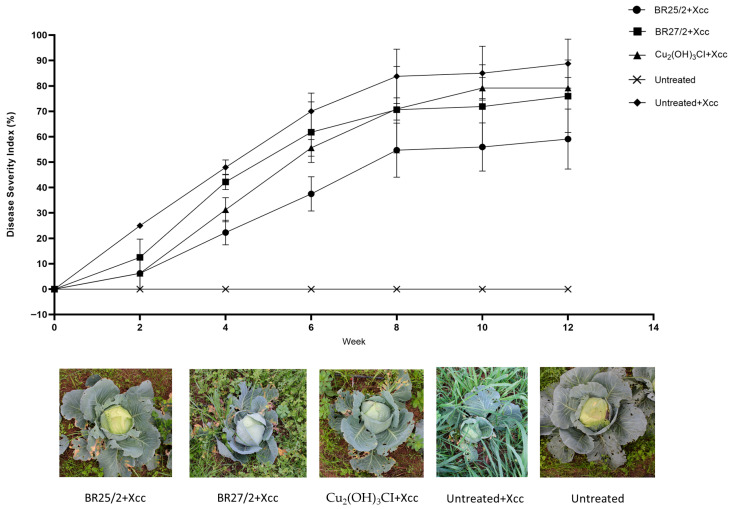
Field trial evaluation of biocontrol efficacy at two distinct locations. The data represents the cumulative disease severity index (%) based on the Townsend–Heuberger conversion. Representative plant images below demonstrate consistent suppression of black rot symptoms across both soil types.

Overall, the ANOVA results across both trials confirm that BR25/2 + Xcc provides a robust and statistically significant reduction in disease severity throughout the 12-week period. Detailed biweekly statistical groupings are available in Table S2 and visualized in Figure 4.

#### 3.3.2. Results of Yield in Field Trials

##### Result of Yield Without Xcc

In the first field trial, the endophytic treatments exhibited a favorable numerical trend concerning plant weight, with BR27/2 (3957.50 ± 1048.50 g) and BR25/2 (3390.00 ± 719.88 g) attaining the highest average weights in comparison to Cu_2_(OH)_3_Cl (2687.50 ± 448.73 g) and the untreated control (2183.33 ± 1071.18 g). Similar patterns were evident in vegetative metrics; BR27/2 recorded the greatest values for plant height (14.75 ± 1.89 cm) and horizontal diameter (89.50 ± 6.38 cm), while BR25/2 exhibited superior measurements in plant width (28.00 ± 1.69 cm) and vertical diameter (70.75 ± 5.03 cm). Despite these numerical enhancements, statistical analysis revealed that all treatments remained statistically indistinguishable from one another (Table 2).

In the second field trial, BR27/2 demonstrated the highest mean weight (1887.50 ± 596.34 g), followed by the untreated group (2386.67 ± 820.72 g), while BR25/2 (1383.33 ± 581.64 g) and the chemical control (1060.00 ± 325.45 g) exhibited lower average weights. This trend was similarly reflected in vegetative parameters, with both the untreated group and BR27/2 presenting the highest measurements for height, width, and diameter. Nevertheless, as noted in the initial trial, Tukey’s post hoc analysis indicated no statistically significant differences among the biocontrol candidates, the chemical control, and the negative control for any measured parameter.

The combined results from the two field trials suggest that while the isolates and chemical control effectively mitigated disease, their influence on final physical yield and growth dimensions under the specific field conditions examined did not achieve statistical significance in comparison to the controls.

Correlation analysis of the two trials revealed strong positive relationships among all morphological characteristics. Fruit weight exhibited a significant correlation with horizontal diameter (r = 0.94) and width (r = 0.91), indicating that weight is fundamentally influenced by the transverse growth of the cabbage. A very strong correlation (r = 0.97) between vertical and horizontal diameters suggested symmetrical fruit development, while fruit height demonstrated a relatively weaker yet positive relationship with other metrics.

**Figure 5:**
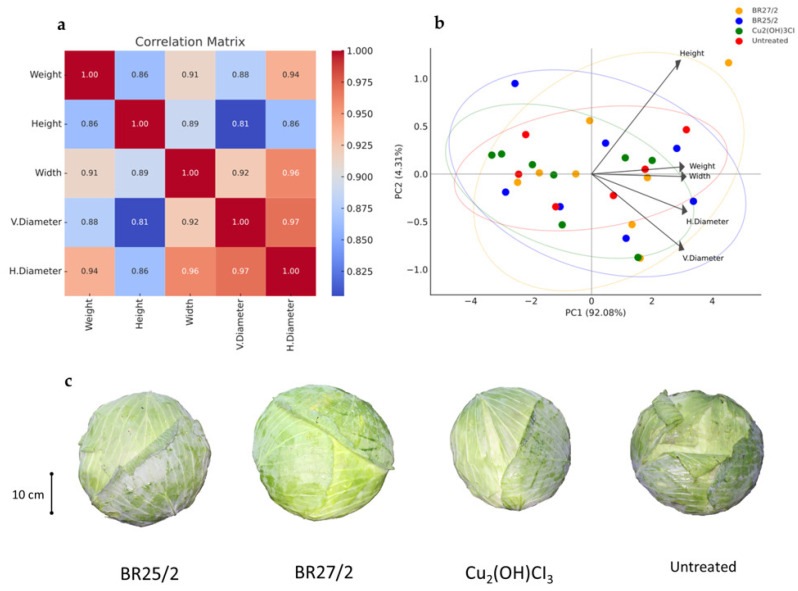
Integration of multivariate statistical analyses and cabbage yield parameters without Xcc. (**a**) Correlation matrix illustrating the relationships between various growth yield parameters. (**b**) Principal Component Analysis (PCA) biplot showing the clustering of isolates and the influence of different variables. (**c**) Representative visual evidence of harvested cabbage heads, demonstrating size and quality in the isolate treatments.

The Principal Component Analysis (PCA) further substantiated these findings, with the first two components accounting for 96.4% of the total variance. PC1 alone explained 92.1% of the differentiation, primarily associated with weight, width, and diameters, which collectively represent overall biomass and fruit size. In contrast, PC2 accounted for only 4.3% of the variance and was more closely related to the height parameter. The PCA scores effectively delineated the treatment groups (Figure 5); the BR25/2 application exhibited superior weight and size characteristics, positioning itself to the right on the PC1 axis, whereas the untreated control clustered in the lower-left quadrant, indicating low weight and size values. While the Cu_2_(OH)_3_Cl treatment displayed a more centralized and compact distribution indicative of homogeneous results, the BR27/2 application exhibited considerable variability among its replicates. These results highlight that PCA effectively distinguishes the performance of the treatments, particularly confirming the superior size parameters associated with the BR25/2 isolate (Figure 5).

##### Result of Yield with Xcc

In the first field trial under Xcc pressure, significant differences in plant weight were observed among the treatments. The BR25/2 + Xcc treatment achieved the highest mean weight (1953.33 ± 747.02 g), while Cu_2_(OH)_3_Cl + Xcc (980.00 ± 383.87 g) and BR27/2 + Xcc (596.67 ± 32.83 g) exhibited intermediate results. The untreated + Xcc group recorded the lowest weight (326.67 ± 133.87 g). Although plant height did not demonstrate statistical significance, other growth parameters, including width, vertical diameter, and horizontal diameter, revealed significant differences. In all these metrics, BR25/2 + Xcc outperformed the other treatments, particularly achieving the highest horizontal diameter (71.33 ± 9.68 cm) compared to the untreated + Xcc control (44.00 ± 1.73 cm).

In the second field trial, BR25/2 + Xcc consistently yielded the highest weight (2342.50 ± 369.15 g) and vertical diameter (61.75 ± 3.20 cm), demonstrating statistically significant differences from the infected control. The chemical control Cu_2_(OH)_3_Cl + Xcc performed comparably to BR25/2 in terms of weight (2116.67 ± 723.56 g) and height (12.33 ± 1.45 cm). Although differences in plant height, width, and horizontal diameter were numerically evident, they did not achieve statistical significance in this trial (*p* > 0.05). Across both experimental seasons, the untreated + Xcc group consistently recorded the lowest values across all measured parameters, underscoring the severity of yield loss in the absence of protective measures.

Overall, the results from both field trials affirm that the BR25/2 isolate exhibited superior efficacy in mitigating yield loss under disease pressure. While Cu_2_(OH)_3_Cl demonstrated moderate effectiveness and BR27/2 yielded lower values, BR25/2 provided the most robust protection for plant biomass and morphological development against Xcc (Table 3).

Correlation analysis indicated that fruit morphological relationships were altered under disease stress. A strong correlation was observed between fruit weight and vertical diameter (r = 0.91), suggesting that mass is predominantly influenced by longitudinal growth. Conversely, the weak correlation between horizontal diameter and height (r = 0.45) implies that these parameters operate as independent variables. Moderate correlations between fruit width and both vertical (r = 0.81) and horizontal (r = 0.61) diameters, along with an r = 0.78 correlation between longitudinal and transverse growth, confirm that these dimensions do not develop concurrently under Xcc pressure.

**Figure 6:**
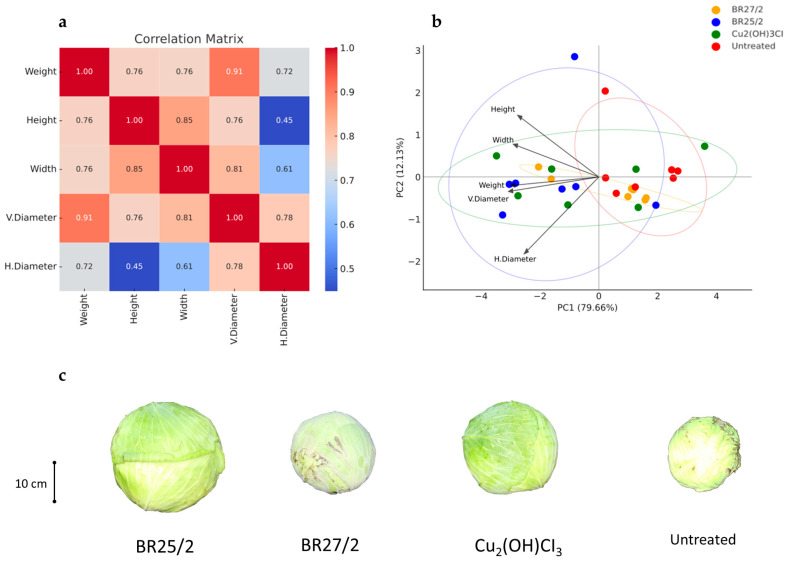
Integration of multivariate statistical analyses and cabbage yield parameters with Xcc. (**a**) Correlation matrix illustrating the relationships between various growth yield parameters. (**b**) Principal Component Analysis (PCA) biplot showing the clustering of isolates and the influence of different variables. (**c**) Representative visual evidence of harvested cabbage heads, demonstrating size and quality in the isolate treatments.

Principal Component Analysis (PCA) accounted for 96.5% of the total variance, with PC1 explaining 92.1% and PC2 4.3%. High loadings of weight, width, and both diameters on PC1 indicated its role as a measure of overall fruit magnitude, while PC2 was specifically associated with height. Treatment distributions within the PCA plane (Figure 6) displayed distinct performance patterns: the BR25/2 + Xcc treatment consistently maintained high magnitude (located on the right side of PC1), whereas the BR27/2 + Xcc treatment exhibited considerable variability. The Cu_2_(OH)_3_Cl + Xcc group demonstrated a compact, homogeneous effect, while the untreated + Xcc control clustered in the lower-left quadrant, characterized by the lowest growth parameters. These findings illustrate that PCA effectively differentiates treatment efficacy, underscoring the superior capacity of BR25/2 + Xcc to sustain fruit size under disease stress (Figure 6).

### 3.4. Results of in Molecular Analysis

#### 3.4.1. Results of DNA Barcoding in the 16S rRNA Region

At the conclusion of the study, oligonucleotide sequences were extracted from the 16S rRNA barcoding region of the two most successful isolates. The bidirectionally obtained sequences were assembled into contig sequences and subsequently subjected to a search in the NCBI database. According to the 16S rRNA gene sequencing results, the two most effective isolates were identified, BR25/2 as *Pseudomonas synxantha* (Ehrenberg 1840) Holland 1920 (98.05% similarity; NCBI Accession No: PX307947, Reference No: KC834335) and BR27/2 as *Pseudomonas baetica* López 2012 (97.72% similarity; NCBI Accession No: PX308179, Reference No: MH012192) (Table S3).

#### 3.4.2. Results of Whole-Genome Sequencing of the Most Successful Isolate

**Figure 7:**
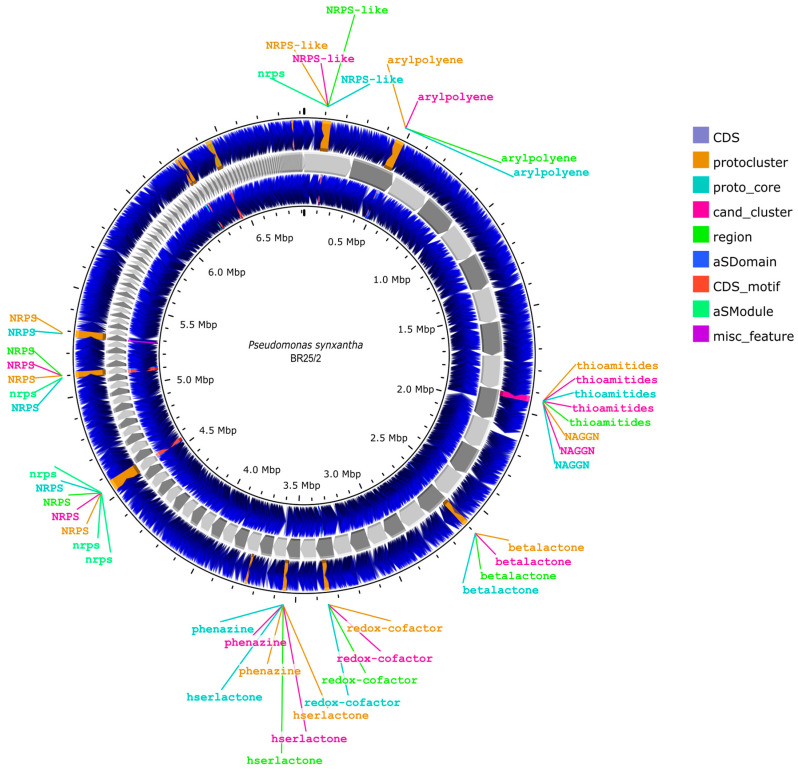
Schematic diagram of the annotation of the metabolite genes possessed by *Pseudomonas synxantha* BR25/2. CDS: Coding Sequence–protein-coding gene region, protocluster: Potential biosynthetic gene cluster, proto_core: Core region of a cluster, cand_cluster: Candidate gene cluster, region: Predicted genomic region, aSDomain: Enzymatic domain detected by AntiSMASH. CDS motif: Motifs within coding sequences. aSModule: AntiSMASH module. misc_feature: Miscellaneous features (various descriptions).

The raw whole-genome sequencing (WGS) file obtained from Genoks Genetic Centre Company underwent bioinformatic analysis, as detailed in Section 2.5. Gene annotation for the identification of metabolite presence was conducted using the antiSMASH software (v 6.1.1) (Figure 7).

Genome mining of *P. synxantha* BR25/2 (BioProject Number: PRJNA1320526, BioSample Number: SAMN51152109, Accession Number: JBQWRJ000000000) utilizing AntiSMASH resulted in the identification of a total of eleven biosynthetic gene clusters (BGCs). These clusters were distributed across various contigs, with sizes ranging from approximately 2.2 kb (Region 168.1) to greater than 55 kb (Region 37.1). The majority of these clusters (8 out of 11) were classified within the non-ribosomal peptide synthetase (NRPS) or NRPS-like categories, highlighting the strain’s considerable capacity for non-ribosomal peptide biosynthesis. Among the well-characterized clusters, Region 1.1 (87,683–129,452 bp) encoded an NRPS-like gene cluster exhibiting 87% similarity to coronatine, whereas Region 14.1 (4225–31,086 bp) was identified as a betalactone cluster with 13% similarity to fengycin. Additionally, Region 20.1 (47,937–70,084 bp) encoded a redox-cofactor cluster associated with lankacidin C (13% similarity), indicating a hybrid NRPS/PKS system. Furthermore, Region 22.1 (62,502–80,713 bp) was designated for phenazine biosynthesis, displaying similarity to streptophenazine B/C.

Three distinct pyoverdine siderophore clusters were identified in Regions 37.1 (3558–55,110 bp), 49.1 (1–26,074 bp), and 105.1 (1–12,681 bp), consistent with the high siderophore-producing capability of BR25/2. Additional clusters included those associated with arylpolyene (Region 2.1; 173,438–217,013 bp; 45% similarity to APE Vf), coelibactin-related (Region 54.1; 1–34,016 bp), fragin-like (Region 94.1; 1–17,283 bp), and bicornutin A1/A2-related (Region 168.1; 1–2273 bp). (Table S4).

In summary, the AntiSMASH analysis provides compelling evidence that *P. synxantha* BR25/2 possesses a functionally diverse repertoire of BGCs, encompassing clusters that encode siderophores (pyoverdine), phenazines, and lipopeptide-like compounds (coronatine- and fengycin-related). The genomic evidence, particularly the presence of multiple extensive NRPS/PKS clusters, establishes a solid molecular foundation for the biocontrol and plant growth-promoting traits observed in experimental assays.

## 4. Discussion

Sustainable management of black rot disease requires effective biological alternatives, especially as Xcc continues to develop resistance against traditional copper-based chemical controls. The present study offers a detailed characterization of 65 bacterial isolates collected from several cabbage-growing regions, emphasizing their biochemical characteristics, PGPR properties, and antagonistic activities against Xcc. The predominance of Gram-negative isolates (60.0%) over Gram-positive ones (40.0%) reflects a trend commonly observed in plant-associated microbiomes, wherein Proteobacteria, particularly *Pseudomonas* spp., are extensively distributed in rhizosphere and phyllosphere environments [41]. This distribution underlines the ecological superiority of Gram-negative bacteria in under field conditions. The isolates showed unstable distribution across the locations, with Menemen having the largest proportion (33.3%). Such variation may be attributed to differences in soil type, agricultural practices, and microclimatic conditions, which are known to influence diversity [42]. The comparison with a low number of isolates from Kınık and Ödemiş suggests either reduced microbial diversity or suboptimal sampling conditions in the areas. These results highlight the importance of regional heterogeneity in microbial diversity structures and the necessity for extensive geographic sampling when identifying biocontrol candidate microorganisms. Among the evaluated PGPR traits, siderophore production was the most common trait, detected in 75.4% of the isolates. The ability to chelate iron confers a dual advantage: enhancing plant nutrition while limiting pathogen microorganism growth by denying them essential micronutrients [43]. Isolates BR29/2, BR28/3, and BR30/1 exhibited the most significant siderophore activity, demonstrating their potential as effective rivals in iron-limited environments typical of plant rhizospheres. Phosphatase activity was discovered in 27.7% of the isolates, with BR10/1, BR36/2, and BR37/2 displaying the highest activity levels. Although less common, this trait is especially valuable in phosphorus-deficient soils, where microbial solubilization enhances nutrient availability and promotes plant growth [44]. Particularly, all isolates demonstrated indole-3-acetic acid (IAA) production, underlying the ubiquity of auxin biosynthesis among plant associated bacteria. Elevated levels of IAA production in isolates BR37/2, BR36/2, and BR38/3 suggest their strong potential for root system modulation, seedling establishment, and improved stress tolerance in cabbage plants [45]. Otherwise, none of the isolates produced hydrogen cyanide (HCN), a finding that diverges from several reports in which *Pseudomonas fluorescens* (Trevisan 1889) Migula 1895 and *Bacillus* spp. exhibited HCN-mediated pathogen prevention [46]. The absence of HCN production in this study demonstrates that antagonism is mainly mediated by siderophores, hydrolytic enzymes, or other secondary metabolites rather than volatile toxins. In vitro assays showed that 32.3% of the isolates (21 strains) displayed antagonistic activity against Xcc. The most active isolates (BR33/2, BR44/1, and BR7/1) exhibited inhibition zones ranging from 8.55 to 9.93 mm. These results are consistent with previous studies on *B. velezinsis* and *Paenibacillus peoriae* (Burkholder 1996) (Montefusco et al. 1993 emend). Heyndrickx et al. 1996 demonstrated similar levels of suppression against *Xanthomonas* spp. through antibiosis [47]. Taken together, the PGPR and antagonistic traits underly the multifunctional potential of the isolates. The weighted scoring approach made possible the identification of 10 promising isolates (BR28/3, BR27/1, BR7/1, BR27/2, BR10/1, BR44/1, BR33/2, BR28/2, BR25/2, and BR34/1) for subsequent *in planta* assays. This integrated selection method aligns with recent research recommendations, which underline the importance of combining biochemical, PGPR, and antagonistic data to enhance the possibility of success in *in planta* and field conditions [48,49].

*In planta* assays provide a valuable understanding of the performance of microbial biological control agents under real environmental effects, as opposed to in vitro conditions. In this study, the isolate BR25/2 demonstrated efficacy levels of up to 91.62% in reducing disease severity. Such pronounced effectiveness aligns with prior research proving the strong biocontrol potential of species such as *Bacillus subtilis* Cohn 1872 and *Pseudomonas fluorescens* against *Xanthomonas* spp. pathogens. For example, Mishra et al. [50] reported that *Pseudomonas fluorescens* strains could reduce black rot disease in cabbage, while Massomo et al. [51] found that isolates of *Bacillus* spp. could mitigate disease symptoms caused by Xcc. Additionally, other isolates, including BR10/1 and BR44/1, achieved significant efficacy levels, approaching or exceeding 70% in certain trials. Otherwise, BR7/1 exhibited poor performance and, in some instances, even exacerbated disease severity. This variability underlines the necessity for isolate-level screening and may reflect microbial antagonism or incompatibility with the host microbiome [16]. The conversion of laboratory success into field performance represents a principal problem in biological control [52]. In this study, BR25/2 proved its efficacy under field conditions, demonstrating statistically significant reductions in disease severity, especially during periods of high disease pressure (Weeks 4–12). For example, in the first trial, disease severity in the untreated + Xcc group reached 88.75% by Week 12, while BR25/2 reduced it to 59.06%, reflecting an efficacy of 33.5%. Notably, the performance of Cu_2_(OH)_3_Cl, a chemical control agent, was lower or statistically comparable to that of BR25/2 across most time points. This result supports the literature suggesting microbial biocontrol agents as possible alternatives to chemical pesticides, especially in light of the environmental and health risks related to the latter [53,54]. Although BR27/2 demonstrated a good performance in early field trials, its efficacy was reduced significantly by the end of the harvest, and it was in the same group as the untreated + xcc group in the later stages. This variability suggests that environmental conditions or microbial competition may help in understanding the performance of certain biocontrol agents over time. Beyond disease suppression, BR25/2 also favorably affected yield-related parameters. Under both disease-free and disease-present conditions, BR25/2 consistently yielded higher mean values for weight, width, and fruit diameter. For example, in disease conditions, BR25/2 + Xcc treatments resulted in cabbage heads weighing up to 2342.50 g, significantly increasing over both the positive control and chemical control treatments. This superior performance may be due to plant growth-promoting rhizobacteria mechanisms, including phytohormone production (e.g., IAA), phosphate solubilization, or nutrient competition [55,56]. Furthermore, BR25/2 distinctly clustered in Principal Component Analysis (PCA), indicating a significant impact on morphological traits. Correlation analyses confirmed strong relationships between yield and dimensions (horizontal diameter and width), proving the hypothesis that BR25/2 may influence fruit biomass accumulation. This aligns with previous studies mentioning biocontrol agent inoculation to improve plant growth and productivity [57].

In the molecular tests, the most effective bacterial isolate, BR25/2, was identified as *Pseudomonas synxantha* through 16S rRNA gene sequencing, displaying 98.05% similarity to known strains. This study identifies *P. synxantha* BR25/2 as the most successful bacterium. *P. synxantha* is known to have significant potential in agricultural and environmental applications, especially for its plant growth-promoting properties and biocontrol capabilities. The bacterium shows strong antagonistic activity against plant pathogens, such as *Pseudomonas syringae* pv. *actinidiae* Takikawa 1989, through the synthesis of non-ribosomal peptides (NRPs) and other bioactive compounds [58]. Furthermore, it produces volatile organic compounds (VOCs) that reduce the growth of fungal pathogens, including *Cadophora luteo-olivacea* and *Botrytis cinerea*, thereby positioning it as a potential biocontrol agent for postharvest diseases in kiwifruit [59]. This study marks the first report of *P. synxantha* as a biocontrol agent against *Xanthomonas campestris* pv. *campestris*. The broad-spectrum antagonistic activity of *P. synxantha* BR25/2 suggests its potential utility across a diverse range of cruciferous crops. Since Xcc is the primary agent of black rot in nearly all Brassicaceae, including broccoli, cauliflower, and radish, the systemic colonization and bioactive metabolite production of BR25/2 are expected to provide similar protective benefits in these hosts, offering a versatile tool for integrated pest management.

A genome-wide analysis of *P. synxantha* BR25/2 showed a broad and diverse array of secondary metabolite biosynthetic gene clusters (BGCs), supporting the experimental results of its high biocontrol efficacy. A total of eleven BGCs were identified, of which eight were classified as non-ribosomal peptide synthetase (NRPS) or NRPS-like, indicating significant biosynthetic potential for bioactive compounds. NRPS-derived metabolites are well known for their structural diversity and antimicrobial potency, attributable to the inclusion of non-standard amino acids and cyclic structures [60]. In addition to conventional NRPS clusters, hybrid NRPS-polyketide synthase (PKS) clusters were also detected, including one that showed similarity to lankacidin C biosynthesis. Hybrid NRPS–PKS systems are related to the synthesis of structurally complex antimicrobial compounds and are frequently found in strains exhibiting strong biocontrol potential [61,62]. A particularly important result is the identification of a coronatine-like NRPS gene cluster with 87% similarity to the coronatine biosynthesis pathway of *Pseudomonas syringae* van Hall (1902). While coronatine functions as a phytotoxin in pathogenic Pseudomonas strains by imitating jasmonic acid to suppress host defenses [63], recent investigations have indicated its potential role in inducing systemic resistance when expressed in non-pathogenic backgrounds [64,65]. This dual function raises the possibility that the BR25/2 strain may utilize coronatine analogs to adjust plant immune responses beneficially, thereby contributing to biocontrol activity beyond mere antagonism. The genome analysis also demonstrated a phenazine biosynthesis gene cluster, which encodes redox-active compounds’ ability to inhibit a broad spectrum of soilborne pathogens through the generation of reactive oxygen species [66,67]. Phenazines also promote ecological conditions by enhancing root colonization and biofilm formation [68]. Another significant result is the identification of a fengycin-like betalactone gene cluster. Fengycin, mainly produced by *Bacillus* spp., is well known for its potent antifungal properties, especially against fungi [69]. The presence of a fengycin-like gene cluster in *P. synxantha* is atypical and may reflect horizontal gene transfer augmenting the strain’s spectrum of antimicrobial activity. In addition to antimicrobial metabolite clusters, BR25/2 contained three distinct pyoverdine siderophore clusters, indicating a strong iron acquisition system. Pyoverdines make iron uptake possible under limiting conditions while restricting iron availability to competing microbes, thereby contributing to pathogen suppression through nutrient competition [70,71]. The redundancy of siderophore systems gives it a highly competitive phenotype that may confer advantages within the rhizosphere. Furthermore, additional BGCs related to arylpolyene, coelibactin, fragin, and bicornutin biosynthesis were identified. While these clusters remain incompletely characterized, prior studies mention roles in oxidative stress protection, quorum sensing and interspecies competition [72]. Collectively, the genome analysis of BR25/2 discovered multifunctional biocontrol metabolites, including direct pathogen inhibition, competition for micronutrients, and adjustment of host immunity. This combination of features may explain the consistent and superior performance of BR25/2 in both *in planta* and field trials. The relatively low similarity of its 16S rRNA gene and distinct genomic features suggest that BR25/2 may represent a novel ecotype or even a new taxon within the *P. synxantha* group, pending further genome-wide comparisons utilizing average nucleotide identity or digital DNA–DNA hybridization [73]. From a practical perspective, the extensive biosynthetic potential of BR25/2, particularly its ability to produce coronatine-like, fengycin-like, and phenazine metabolites, positions it as a potential candidate for development as a microbial solution.

## 5. Conclusions

This study presents a comprehensive evaluation of bacterial isolates exhibiting biocontrol potential against *Xanthomonas campestris* pv. *campestris* (Xcc), the causative agent of black rot in cruciferous crops. Employing a multi-phase approach that includes biochemical profiling, plant growth-promoting rhizobacteria trait analysis, in vitro antagonism assays, *in planta* evaluations, and genome-wide characterization, the isolate BR25/2 (identified as *P. synxantha*) emerges as a highly effective biocontrol agent. Isolate BR25/2 consistently demonstrated disease suppression, enhanced plant growth parameters, and competitive performance in field trials, surpassing conventional copper-based treatments in several instances. Its biocontrol efficacy is driven by a diverse array of biosynthetic gene clusters, including those responsible for the production of siderophores, phenazines, and coronatine-like compounds, indicating complex and powerful antimicrobial metabolites. This study provides the first report of *P. synxantha* as a biocontrol agent against Xcc, thereby expanding the identified ecological role of this species and contributing novel insights to the domain of plant–microbe interactions. The integration of phenotypic data with genomic evidence increases the candidacy of BR25/2 for development as a microbial solution. Additionally, its low sequence identity to known strains suggests potential taxonomic novelty, prompting more phylogenomic investigations. Future directions will focus on large-scale fermentation and formulation development to ensure field stability and shelf life. We aim to initiate the official registration process of *P. synxantha* BR25/2 as a commercial bioproduct, involving rigorous toxicological and environmental safety assessments. In light of increasing chemical resistance, *P. synxantha* BR25/2 represents a promising, eco-friendly alternative for managing black rot disease and enhancing sustainable cabbage productivity globally.

## Figures and Tables

**Table 1:** Table of statistical results from the *in planta* assay conducted at 95% confidence.

Isolates	*In Planta* Assay 1		*In Planta* Assay 2		*In Planta* Assay 3	
	Disease Severity Index (%)	Efficacy (%)	Disease Severity Index (%)	Efficacy (%)	Disease Severity Index (%)	Efficacy (%)
**BR7/1**	38.57 ± 5.46 a*	−0.89	18.66 ± 4.62 ab	32.90	31.05 ± 4.25 b	61.84
**BR10/1**	16.54 ± 4.32 bcd	56.74	14.46 ± 3.86 b	48.00	14.00 ± 3.29 cd	82.79
**BR25/2**	10.82 ± 4.07 de	71.70	9.92 ± 4.07 bc	64.33	6.82 ± 3.68 de	91.62
**BR27/1**	23.88 ± 4.32 bc	37.54	12.84 ± 3.86 b	53.83	20.57 ± 3.47 bc	74.72
**BR27/2**	15.66 ± 4.07 cd	59.04	13.87 ± 3.86 b	50.13	12.14 ± 4.66 cd	85.08
**BR28/1**	28.12 ± 4.07 ab	26.45	14.16 ± 4.07 b	49.08	9.26 ± 3.29 de	88.62
**BR28/2**	23.45 ± 4.61 bc	38.66	15.69 ± 4.62 b	43.58	21.25 ± 4.25 bc	73.88
**BR33/2**	24.37 ± 3.86 bc	36.25	14.07 ± 3.86 b	49.41	12.76 ± 3.94 cd	84.32
**BR34/1**	23.42 ± 4.07 bc	38.74	11.21 ± 3.86 b	59.69	12.84 ± 3.47 cd	84.22
**BR44/1**	14.32 ± 4.07 cd	62.54	17.97 ± 4.07 ab	35.38	10.27 ± 3.94 cd	87.38
**Untreated**	0.00 ± 0.00 e	100.00	0.00 ± 0.00 c	100.00	0.00 ± 0.00 e	100.00
**Untreated + Xcc**	38.23 ± 3.86 a	0.00	27.81 ± 3.86 a	0.00	81.37 ± 3.29 a	0.00
**ANOVA**	Cv: 58.40; F: 6.95; *p*: 2.11 × 10^−8^		Cv: 100.80; F: 2.69; *p*: 0.00445		Cv: 43.10; F: 39.46; *p*: 2.2 × 10^−16^	

* The difference between characters with the same letter value is statistically insignificant according to the Tukey test conducted at 95%.

**Table 2:** Table of statistical results from the field trial according to yield without disease conducted at 95% confidence.

Parameters	Field Trials	BR25/2	BR27/2	Cu_2_(OH)_3_CI	Untreated	ANOVA
**Weight (g)**	**1. Field**	3390.00 ± 719.88 a*	3957.50 ± 1048.50 a	2687.50 ± 448.73 a	2183.33 ± 1071.18 a	Cv: 51.30; F: 1.85; *p*: 0.192
	**2. Field**	1383.33 ± 581.50 a	1887.50 ± 596.34 a	1060.00 ± 325.45 a	2386.67 ± 820.59 a	Cv: 60.70; F: 1.42; *p*: 0.285
**Height (cm)**	**1. Field**	13.75 ± 1.65 a	14.75 ± 1.89 a	13.25 ± 1.38 a	12.33 ± 2.85 a	Cv: 21.90; F: 2.41; *p*: 0.117
	**2. Field**	11.00 ± 1.73 a	11.50 ± 1.19 a	9.75 ± 0.48 a	13.00 ± 1.15 a	Cv: 19.20; F: 0.95; *p*: 0.447
**Width (cm)**	**1. Field**	28.00 ± 1.69 a	28.00 ± 2.04 a	25.75 ± 1.60 a	24.33 ± 2.96 a	Cv: 14.30; F: 1.98; *p*: 0.171
	**2. Field**	21.33 ± 1.85 a	23.00 ± 1.78 a	18.25 ± 1.18 a	24.00 ± 2.64 a	Cv: 18.70; F: 1.15; *p*: 0.369
**Vertical** **diameter (cm)**	**1. Field**	70.75 ± 5.03 a	69.75 ± 2.92 a	68.75 ± 3.06 a	61.00 ± 8.33 a	Cv: 11.90; F: 2.12; *p*: 0.151
	**2. Field**	50.67 ± 6.49 a	59.25 ± 5.34 a	50.25 ± 2.66 a	63.67 ± 6.44 a	Cv: 19.20; F: 1.28; *p*: 0.326
**Horizontal** **diameter (cm)**	**1. Field**	88.75 ± 6.10 a	89.50 ± 6.38 a	86.75 ± 3.90 a	76.00 ± 10.58 a	Cv: 12.60; F: 2.34; *p*: 0.125
	**2. Field**	65.33 ± 7.33 a	71.00 ± 5.25 a	61.25 ± 3.20 a	77.00 ± 8.08 a	Cv: 16.30; F: 1.04; *p*: 0.409

* The difference between characters with the same letter value is statistically insignificant according to the Tukey test conducted at 95%.

**Table 3:** Table of statistical results from the field trial according to yield with disease conducted at 95% confidence.

Parameters	Field Trials	BR25/2 + Xcc	BR27/2 + Xcc	Cu_2_(OH)_3_CI + Xcc	Untreated + Xcc	ANOVA
**Weight (g)**	**1. Field**	1953.33 ± 747.02 a*	596.67 ± 32.83 ab	980.00 ± 398.06 ab	326.67 ± 87.43 b	Cv: 104.50; F: 4.85; *p*: 0.019
	**2. Field**	2342.50 ± 369.15 a	1260.00 ± 361.82 ab	2116.67 ± 723.56 a	545.00 ± 102.67 b	Cv: 68.30; F: 3.98; *p*: 0.035
**Height (cm)**	**1. Field**	10.67 ± 1.86 a	8.67 ± 0.33 a	9.50 ± 0.96 a	10.00 ± 1.00 a	Cv: 18.50; F: 2.14; *p*: 0.148
	**2. Field**	12.00 ± 0.71 a	10.50 ± 1.19 ab	12.33 ± 1.45 a	9.00 ± 0.41 b	Cv: 19.80; F: 3.88; *p*: 0.087
**Width (cm)**	**1. Field**	23.67 ± 3.93 a	16.67 ± 0.33 a	19.25 ± 2.93 a	17.67 ± 5.17 a	Cv: 29.20; F: 2.91; *p*: 0.078
	**2. Field**	22.25 ± 0.85 a	20.25 ± 2.21 a	22.67 ± 3.38 a	18.25 ± 1.75 a	Cv: 17.40; F: 2.45; *p*: 0.113
**Vertical** **diameter (cm)**	**1. Field**	58.67 ± 6.36 a	48.00 ± 0.58 ab	49.50 ± 7.69 ab	38.33 ± 2.03 b	Cv: 21.90; F: 3.65; *p*: 0.046
	**2. Field**	61.75 ± 3.20 a	54.25 ± 3.64 ab	59.00 ± 7.02 ab	46.75 ± 2.87 b	Cv: 15.60; F: 3.55; *p*: 0.048
**Horizontal** **diameter (cm)**	**1. Field**	71.33 ± 9.68 a	55.67 ± 0.88 ab	58.00 ± 9.90 ab	44.00 ± 1.00 b	Cv: 25.50; F: 4.12; *p*: 0.032
	**2. Field**	61.25 ± 13.73 a	65.50 ± 4.77 a	71.00 ± 9.50 a	56.25 ± 4.37 a	Cv: 25.20; F: 3.12; *p*: 0.065

* The difference between characters with the same letter value is statistically insignificant according to the Tukey test conducted at 95%.

## Data Availability

Data is contained within the article or supplementary material.

## Supplementary Materials

The following supporting information can be downloaded at: https://www.mdpi.com/article/10.3390/life16040647/s1, Table S1: Results of Biochemical and In vitro PGPR Tests for Isolated Candidate Strains; Table S2: Table of statistical results from the field trials conducted at 95%confidence.; Table S3: Molecular Diagnosis and NCBI reference numbers of successful isolates of bacteria; Table S4: Predicted biosynthetic gene cluster in *Pseudomonas synxantha* BR25/2.

## Abbreviations

The following abbreviations are used in this manuscript:

ANOVA	Analysis of Variance
BGC	Biosynthetic Gene Cluster
CAS	Chrome Azurol S
HCN	Hydrogen cyanide
IAA	Indole-3-acetic acid
NRPS	Non-ribosomal peptide synthetase
OD600	Optical Density at 600 nm
PCA	Principal Component Analysis
PCR	Polymerase Chain Reaction
PGPR	Plant Growth-Promoting Rhizobacteria
rRNA	Ribosomal RNA
T3SS	Type III secretion system
TAE	Tris–Acetate–EDTA
VOCs	Volatile Organic Compounds
WGS	Whole-Genome Sequencing
Xcc	*Xanthomonas campestris* pv. *campestris*
