# Supplementary material for: Biocontrol Efficacy and Genomic Basis of Endophytic Bacteria Against Xanthomonas campestris pv. campestris in Cabbage
Source: Life (Basel). 2026 Apr 11;16(4):647. doi: 10.3390/life16040647 (PMC13117714; doi:10.3390/life16040647)
Supplement: Supplementary file 1 [file life-16-00647-s001.zip › Table S1.pdf]

**Table S1**. Results of Biochemical and *In vitro* PGPR Tests for Isolated Candidate Strains

| No | Isolate Code | Location | Latitude | Longitude | *In vitro* Assay | | | | | | | | | |
|---|---|---|---|---|---|---|---|---|---|---|---|---|---|---|
| | | | | | Biochemical Test | | | | | PGPR Test | | | | |
| | | | | | Gr[1] | Fl[2] | Pec[3] | HR[4] | 37ºC[5] | Sid[6] | PhA[7] | IAA[8] | HCN[9] | BioCo[10] |
| 1 | BR1/1 | Torbalı | 38.1102 | 27.4258 | - | - | - | - | - | 0.84 | 0.00 | 32.52 | - | 0.00 |
| 2 | BR2/1 | Torbalı | 38.1210 | 27.5187 | + | - | - | - | - | 0 | 0.00 | 38.91 | - | 0.00 |
| 3 | BR3/1 | Torbalı | 38.1248 | 27.5511 | + | - | - | - | - | 0 | 0.00 | 19.07 | - | 0.00 |
| 4 | BR4/1 | Bayındır | 38.1612 | 27.5129 | - | - | - | - | - | 4.7 | 2.41 | 46.53 | - | 0.00 |
| 5 | BR7/1 | Bayındır | 38.1652 | 27.5228 | - | + | - | - | - | 11.09 | 0.00 | 13.62 | - | 8.55 |
| 6 | BR8/1 | Bayındır | 38.1590 | 27.5382 | - | - | - | - | - | 5.35 | 3.59 | 39.57 | - | 0.00 |
| 7 | BR9/1 | Bayındır | 38.1542 | 27.5430 | + | - | - | - | - | 12.37 | 0.00 | 33.74 | - | 0.00 |
| 8 | BR9/2 | Bayındır | 38.1542 | 27.5430 | - | - | - | - | - | 7.43 | 1.09 | 16.81 | - | 0.00 |
| 9 | BR9/3 | Bayındır | 38.1542 | 27.5430 | - | - | - | - | - | 10.11 | 0.00 | 32.90 | - | 2.71 |
| 10 | BR10/1 | Ödemiş | 38.3323 | 28.0641 | - | - | - | - | - | 10.22 | 8.92 | 3.37 | - | 4.60 |
| 11 | BR11/1 | Selçuk | 38.0178 | 27.4546 | + | - | - | - | - | 0 | 0.00 | 2.61 | - | 0.00 |
| 12 | BR11/2 | Selçuk | 38.0178 | 27.4546 | + | - | - | - | - | 0 | 0.00 | 2.52 | - | 0.00 |
| 13 | BR11/3 | Selçuk | 38.0178 | 27.4546 | - | - | - | - | - | 0 | 0.00 | 2.24 | - | 5.76 |
| 14 | BR17/1 | Selçuk | 38.0062 | 27.4253 | - | - | - | - | - | 0 | 0.00 | 13.05 | - | 0.00 |
| 15 | BR17/2 | Selçuk | 38.0062 | 27.4253 | - | - | - | - | - | 7.02 | 0.00 | 3.37 | - | 3.96 |
| 16 | BR17/3 | Selçuk | 38.0062 | 27.4253 | - | + | - | - | - | 8.57 | 0.00 | 9.29 | - | 2.64 |
| 17 | BR17/4 | Selçuk | 38.0062 | 27.4253 | + | - | - | - | - | 2.35 | 0.00 | 33.93 | - | 5.87 |
| 18 | BR17/5 | Selçuk | 38.0062 | 27.4253 | + | - | - | - | - | 0 | 0.00 | 3.27 | - | 0.00 |
| 19 | BR17/6 | Selçuk | 38.0062 | 27.4253 | - | - | - | - | - | 0 | 0.00 | 0.83 | - | 0.00 |
| 20 | BR18/1 | Menemen | 38.6120 | 27.1051 | + | - | - | - | - | 5.98 | 0.00 | 4.40 | - | 5.68 |
| 21 | BR18/2 | Menemen | 38.6120 | 27.1051 | + | - | - | - | - | 0 | 0.91 | 3.56 | - | 0.00 |
| 22 | BR18/3 | Menemen | 38.6120 | 27.1051 | - | - | - | - | - | 8.08 | 0.85 | 5.53 | - | 0.00 |
| 23 | BR20/1 | Menemen | 38.6231 | 27.1370 | + | - | - | - | - | 0 | 0.00 | 4.78 | - | 0.00 |
| 24 | BR20/2 | Menemen | 38.6231 | 27.1370 | - | + | - | - | - | 6.01 | 3.06 | 12.11 | - | 4.44 |
| 25 | BR23/1 | Menemen | 38.6272 | 27.1297 | - | - | - | - | - | 2.03 | 1.96 | 6.56 | - | 0.00 |
| 26 | BR23/2 | Menemen | 38.6272 | 27.1297 | + | - | - | - | - | 1.22 | 1.66 | 11.08 | - | 0.00 |
| 27 | BR25/1 | Menemen | 38.6178 | 27.1441 | - | + | - | - | - | 11.07 | 0.00 | 29.51 | - | 0.00 |
| 28 | BR25/2 | Menemen | 38.6178 | 27.1441 | - | + | - | - | - | 8.99 | 0.95 | 41.45 | - | 6.81 |
| 29 | BR27/1 | Menemen | 38.5937 | 27.0872 | - | + | - | - | - | 13.37 | 4.65 | 10.42 | - | 6.97 |
| 30 | BR27/2 | Menemen | 38.5937 | 27.0872 | + | - | - | - | - | 0 | 0.00 | 2.99 | - | 0.00 |
| 31 | BR28/1 | Menemen | 38.6480 | 27.0860 | - | - | - | - | - | 9.72 | 0.00 | 14.65 | - | 0.00 |
| 32 | BR28/2 | Menemen | 38.6480 | 27.0860 | - | + | - | - | - | 6.76 | 0.00 | 34.21 | - | 8.44 |

| | | | | | | | | | | | | | | |
|---|---|---|---|---|---|---|---|---|---|---|---|---|---|---|
| 33 | BR28/3 | Menemen | 38.6480 | 27.0860 | - | + | - | - | - | 17.03 | 0.00 | 19.26 | - | 7.46 |
| 34 | BR29/1 | Menemen | 38.6521 | 27.0860 | - | + | - | - | - | 2.54 | 2.02 | 30.17 | - | 0.00 |
| 35 | BR29/2 | Menemen | 38.6521 | 27.0860 | - | + | - | - | - | 19.86 | 0.00 | 13.71 | - | 0.00 |
| 36 | BR29/3 | Menemen | 38.6521 | 27.0860 | - | + | - | - | - | 0 | 0.00 | 0.92 | - | 0.00 |
| 37 | BR29/4 | Menemen | 38.6521 | 27.0860 | - | + | - | - | - | 10.91 | 0.00 | 13.15 | - | 0.00 |
| 38 | BR29/5 | Menemen | 38.6521 | 27.0860 | - | - | - | - | - | 9 | 0.00 | 0.73 | - | 0.00 |
| 39 | BR29/6 | Menemen | 38.6521 | 27.0860 | - | - | - | - | - | 0 | 0.00 | 6.38 | - | 0.00 |
| 40 | BR29/7 | Menemen | 38.6521 | 27.0860 | + | - | - | - | - | 0 | 0.00 | 11.92 | - | 0.00 |
| 41 | BR29/8 | Menemen | 38.6521 | 27.0860 | - | - | - | - | - | 0 | 0.00 | 0.83 | - | 4.41 |
| 42 | BR30/1 | Foça | 38.6636 | 26.8852 | - | + | - | - | - | 14.37 | 2.31 | 27.44 | - | 0.00 |
| 43 | BR31/1 | Foça | 38.6636 | 26.8852 | + | - | - | - | - | 0 | 0.00 | 1.49 | - | 6.62 |
| 44 | BR32/1 | Foça | 38.6612 | 26.8845 | - | - | - | - | - | 2.25 | 0.00 | 34.21 | - | 0.00 |
| 45 | BR32/2 | Foça | 38.6612 | 26.8845 | + | - | - | - | - | 9.73 | 0.00 | 44.84 | - | 0.00 |
| 46 | BR32/3 | Foça | 38.6612 | 26.8845 | + | - | - | - | - | 3.59 | 0.00 | 4.97 | - | 0.00 |
| 47 | BR33/1 | Foça | 38.6620 | 26.8941 | - | + | - | - | - | 7.08 | 2.94 | 15.50 | - | 7.96 |
| 48 | BR33/2 | Foça | 38.6620 | 26.8941 | + | - | - | - | - | 0.92 | 0.00 | 0.83 | - | 9.93 |
| 49 | BR34/1 | Foça | 38.6582 | 26.8908 | + | - | - | - | - | 1.21 | 0.00 | 1.11 | - | 0.00 |
| 50 | BR35/1 | Foça | 38.6546 | 26.8870 | - | + | - | - | - | 9.27 | 0.00 | 19.17 | - | 0.00 |
| 51 | BR35/2 | Foça | 38.6546 | 26.8870 | + | - | - | - | - | 2.42 | 0.00 | 6.56 | - | 0.00 |
| 52 | BR35/3 | Foça | 38.6546 | 26.8870 | - | - | - | - | - | 14.81 | 0.00 | 6.38 | - | 0.00 |
| 53 | BR36/1 | Bergama | 39.0901 | 27.1282 | + | - | - | - | - | 1.2 | 0.00 | 5.91 | - | 0.00 |
| 54 | BR36/2 | Bergama | 39.0901 | 27.1282 | - | - | - | - | - | 5.49 | 4.12 | 52.55 | - | 0.00 |
| 55 | BR36/3 | Bergama | 39.0901 | 27.1282 | + | - | - | - | - | 10.42 | 0.00 | 26.12 | - | 0.00 |
| 56 | BR37/1 | Bergama | 39.1182 | 27.2259 | + | - | - | - | - | 2.27 | 0.00 | 1.20 | - | 0.00 |
| 57 | BR37/2 | Bergama | 39.1182 | 27.2259 | - | - | - | - | - | 5.84 | 5.18 | 53.30 | - | 0.00 |
| 58 | BR37/3 | Bergama | 39.1182 | 27.2259 | + | - | - | - | - | 4.78 | 0.00 | 19.82 | - | 5.19 |
| 59 | BR38/1 | Bergama | 39.1108 | 27.2345 | + | - | - | - | - | 5.15 | 0.00 | 1.20 | - | 4.46 |
| 60 | BR38/2 | Bergama | 39.1108 | 27.2345 | + | - | - | - | - | 4.96 | 0.00 | 4.31 | - | 1.38 |
| 61 | BR38/3 | Bergama | 39.1108 | 27.2345 | - | - | - | - | - | 4.88 | 1.14 | 48.79 | - | 0.00 |
| 62 | BR39/1 | Bergama | 39.1001 | 27.2304 | - | - | - | - | - | 12.07 | 0.00 | 13.05 | - | 0.00 |
| 63 | BR41/1 | Dikili | 39.0692 | 26.9752 | + | - | - | - | - | 1.05 | 0.00 | 3.56 | - | 0.00 |
| 64 | BR41/2 | Dikili | 39.0692 | 26.9752 | - | - | - | - | - | 1.54 | 0.00 | 11.55 | - | 0.00 |
| 65 | BR44/1 | Kınık | 39.0964 | 27.3304 | - | + | - | - | - | 4.51 | 1.26 | 8.07 | - | 9.19 |

1.**Gr:** Gram test, 2. **Fl:** Floresent pigmentation 3. **Pec:** Pectolytic Activity 3. **HR:** Hypersentitive Reaction on Tobacco, 4. **37ºC**: Devolopment at 37ºC 6. **Sid:** Siderophore production (mm), 7. **PhA:** Phosphatase activity (mm), 8. **IAA:** Indole Acetic Acid Production (µg/ml), 7. **PA:** Chitinolytic Activity (mm), 8. **PA:** Proteolytic Activity (mm),9. HCN: Hydrogen cyanide production, 10. **BioCo**: *In vitro* Biocontrol Assay (mm)
