# Supplementary material for: Biocontrol Efficacy and Genomic Basis of Endophytic Bacteria Against Xanthomonas campestris pv. campestris in Cabbage
Source: Life (Basel). 2026 Apr 11;16(4):647. doi: 10.3390/life16040647 (PMC13117714; doi:10.3390/life16040647)
Supplement: Supplementary file 1 [file life-16-00647-s001.zip › Table S2.pdf]

Table S2. Table of statistical results from the field trials conducted at 95%confidence.

Time Point	Field Trials	Evaluation	BR25/2+xcc	BR27/2+xcc	$Cu_2(OH)_3CI$+xcc	Untreated	Untreated+xcc	ANOVA
**0.week**	**1.Field**	**DSI (%)**	0.00 ± 0.00 a	0.00 ± 0.00 a	0.00 ± 0.00 a	0.00 ± 0.00 a	0.00 ± 0.00 a	
		**Efficacy (%)**	0.00	0.00	0.00	0.00	0.00	
	**2.Field**	**DSI (%)**	0.00 ± 0.00 a	0.00 ± 0.00 a	0.00 ± 0.00 a	0.00 ± 0.00 a	0.00 ± 0.00 a	
		**Efficacy (%)**	0.00	0.00	0.00	0.00	0.00	
**2.week**	**1.Field**	**DSI (%)**	6.25 ± 6.25 b	12.50 ± 7.22 ab	6.25 ± 6.25 b	0.00 ± 0.00 b	25.00 ± 0.00 a	Cv:168.40; F:2.15 ; p:0.147
		**Efficacy (%)**	75.00	50.00	75.00	100.00	0.00	
	**2.Field**	**DSI (%)**	6.25 ± 6.25 b	12.50 ± 7.22 ab	6.25 ± 6.25 b	0.00 ± 0.00 b	25.00 ± 0.00 a	Cv:182.10; F:1.34 ; p:0.308
		**Efficacy (%)**	75.00	50.00	75.00	100.00	0.00	
**4.week**	**1.Field**	**DSI (%)**	22.29 ± 4.85 b	42.19 ± 2.99 a	31.25 ± 4.73 b	0.00 ± 0.00 c	47.92 ± 2.92 a	Cv:48.20; F:0.84 ; p:0.498
		**Efficacy (%)**	53.48	11.96	34.78	100.00	0.00	
	**2.Field**	**DSI (%)**	33.12 ± 1.88 c	40.62 ± 3.29 b	36.25 ± 1.25 bc	0.00 ± 0.00 d	52.81 ± 2.62 a	Cv:35.60; F:0.45 ; p:0.724
		**Efficacy (%)**	37.28	23.08	31.36	100.00	0.00	
**6.week**	**1.Field**	**DSI (%)**	37.50 ± 6.75 b	61.77 ± 11.95 a	55.62 ± 3.29 ab	0.00 ± 0.00 c	70.00 ± 7.14 a	Cv:42.50; F:1.83; p:0.195
		**Efficacy (%)**	46.43	11.76	20.54	100.00	0.00	
	**2.Field**	**DSI (%)**	45.00 ± 4.08 a	49.17 ± 4.04 a	46.25 ± 4.27 a	0.00 ± 0.00 b	55.62 ± 5.62 a	Cv:28.40; F:0.58 ; p:0.637
		**Efficacy (%)**	19.10	11.61	16.85	100.00	0.00	
**8.week**	**1.Field**	**DSI (%)**	54.69 ± 10.64 a	70.62 ± 17.03 a	70.94 ± 4.37 a	0.00 ± 0.00 b	83.75 ± 10.68 a	Cv:38.70; F:3.42 ; p:0.053
		**Efficacy (%)**	34.70	15.67	15.30	100.00	0.00	
	**2.Field**	**DSI (%)**	50.00 ± 2.89 a	53.33 ± 3.27 a	54.38 ± 5.22 a	0.00 ± 0.00 b	62.81 ± 7.40 a	Cv:24.80; F:0.61 ; p:0.619
		**Efficacy (%)**	20.40	15.09	13.43	100.00	0.00	
**10.week**	**1.Field**	**DSI (%)**	55.94 ± 9.48 a	71.88 ± 16.44 a	79.17 ± 4.17 a	0.00 ± 0.00 b	85.00 ± 10.61 a	Cv:35.40; F:3.91 ; p:0.037
		**Efficacy (%)**	34.19	15.44	6.86	100.00	0.00	
	**2.Field**	**DSI (%)**	53.75 ± 4.27 a	62.81 ± 4.58 a	58.65 ± 6.19 a	0.00 ± 0.00 b	68.44 ± 7.49 a	Cv:21.20; F:0.65 ; p:0.596
		**Efficacy (%)**	21.46	8.22	14.31	100.00	0.00	
**12.week**	**1.Field**	**DSI (%)**	59.06 ± 11.81 b	75.94 ± 14.28 ab	79.17 ± 4.17 ab	0.00 ± 0.00 c	88.75 ± 9.66 a	Cv:32.10; F:4.25 ; p:0.029
		**Efficacy (%)**	33.45	14.44	10.80	100.00	0.00	
	**2.Field**	**DSI (%)**	58.75 ± 3.92 a	68.75 ± 4.42 a	68.44 ± 6.56 a	0.00 ± 0.00 b	72.81 ± 7.88 a	Cv:19.50; F:3.82 ; p:0.409
		**Efficacy (%)**	19.31	5.58	6.01	100.00	0.00	

*The difference between characters with the same letter value is statistically insignificant according to the Tukey test conducted at 95%.

** DSI: Disease Severity Index
