# Supplementary material for: Biocontrol Efficacy and Genomic Basis of Endophytic Bacteria Against Xanthomonas campestris pv. campestris in Cabbage
Source: Life (Basel). 2026 Apr 11;16(4):647. doi: 10.3390/life16040647 (PMC13117714; doi:10.3390/life16040647)
Supplement: Supplementary file 1 [file life-16-00647-s001.zip › Table S3.pdf]

Table S3. Molecular Diagnosis and NCBI reference numbers of successful isolates of bacteria

Isolate Code	Bacteria Species	Reference Similarity Rate	Reference Access No.	NCBI Access No.
BR25/2	*Pseudomonas synxantha*	98.05	KC834335	PX307947
BR27/2	*Pseudomonas baetica*	97.72	MH012192	PX308179
