# Supplementary material for: Biocontrol Efficacy and Genomic Basis of Endophytic Bacteria Against Xanthomonas campestris pv. campestris in Cabbage
Source: Life (Basel). 2026 Apr 11;16(4):647. doi: 10.3390/life16040647 (PMC13117714; doi:10.3390/life16040647)
Supplement: Supplementary file 1 [file life-16-00647-s001.zip › Table S4.pdf]

Table S4. Predicted biosynthetic gene cluster in *Pseudomonas synxantha* BR25/2

Region	Type	Desciption	Size (bp)	Most similar known cluster	Similarity (%)
Region 1.1	NRPS-like	NRPS (Non-Ribosomal Peptide Synthetase)-like clusters (similar to NRPS but with incomplete or variant structures).	87.683–129.452	coronatine	87%
Region 2.1	arylpolyene	Aromatic polyene, pigment-like secondary metabolite.	173.438–217.013	APE Vf	45%
Region 14.1	betalactone	Beta-lactone – antibiotic/enzymatic inhibitor-like compound.	4.225–31.086	fengycin	13%
Region 20.1	redox-cofactor	Redox cofactor – small molecule involved in oxidation-reduction processes (e.g., NADH, FADH).	47.937–70.084	lankacidin C	13%
Region 22.1	hserlactone,phenazine	Homoserine lactone – quorum sensing signaling molecule.<br>Phenazine – antimicrobial secondary metabolite.	62.502–80.713	streptophenazine B / streptophenazine C / streptophenazine F / streptophenazine G / streptophenazine H	<10%
Region 37.1	NRPS	*Non-Ribosomal Peptide Synthetase* – enzymes producing diverse peptide secondary metabolites.	3.558–55.110	pyoverdin	<10%
Region 49.1	NRPS	*Non-Ribosomal Peptide Synthetase* – enzymes producing diverse peptide secondary metabolites.	1–26.074	pyoverdin	<10%
Region 54.1	NRPS	*Non-Ribosomal Peptide Synthetase* – enzymes producing diverse peptide secondary metabolites.	1–34.016	coelibactin	<10%
Region 94.1	NRPS-like	NRPS (Non-Ribosomal Peptide Synthetase)-like clusters (similar to NRPS but with incomplete or variant structures).	1–17.283	fragin	<10%
Region 105.1	NRPS	*Non-Ribosomal Peptide Synthetase* – enzymes producing diverse peptide secondary metabolites.	1–12.681	pyoverdin	<10%
Region 168.1	NRPS	*Non-Ribosomal Peptide Synthetase* – enzymes producing diverse peptide secondary metabolites.	1–2.273	bicornutin A1 / bicornutin A2	<10%
Region 1.1	NRPS-like	NRPS (Non-Ribosomal Peptide Synthetase)-like clusters (similar to NRPS but with incomplete or variant structures).	87.683–129.452	coronatine	87%

Region 2.1	arylpolyene	Aromatic polyene, pigment-like secondary metabolite.	173.438–217.013	APE Vf	45%
Region 14.1	betalactone	Beta-lactone – antibiotic/enzymatic inhibitor-like compound.	4.225–31.086	fengycin	13%
Region 20.1	redox-cofactor	Redox cofactor – small molecule involved in oxidation-reduction processes (e.g., NADH, FADH).	47.937–70.084	lankacidin C	13%
Region 22.1	hserlactone,phenazine	Homoserine lactone – quorum sensing signaling molecule.	62.502–80.713	streptophenazine B / streptophenazine C / streptophenazine F / streptophenazine G / streptophenazine H	<10%
Region 37.1	NRPS	*Non-Ribosomal Peptide Synthetase* – enzymes producing diverse peptide secondary metabolites.	3.558–55.110	pyoverdin	<10%
Region 49.1	NRPS	*Non-Ribosomal Peptide Synthetase* – enzymes producing diverse peptide secondary metabolites.	1–26.074	pyoverdin	<10%
Region 54.1	NRPS	*Non-Ribosomal Peptide Synthetase* – enzymes producing diverse peptide secondary metabolites.	1–34.016	coelibactin	<10%
Region 94.1	NRPS-like	NRPS (Non-Ribosomal Peptide Synthetase)-like clusters (similar to NRPS but with incomplete or variant structures).	1–17.283	fragin	<10%
Region 105.1	NRPS	*Non-Ribosomal Peptide Synthetase* – enzymes producing diverse peptide secondary metabolites.	1–12.681	pyoverdin	<10%
Region 168.1	NRPS	*Non-Ribosomal Peptide Synthetase* – enzymes producing diverse peptide secondary metabolites.	1–2.273	bicornutin A1 / bicornutin A2	<10%
